# Indomethacin protects rats from neuronal damage induced by traumatic brain injury and suppresses hippocampal IL-1β release through the inhibition of Nogo-A expression

**DOI:** 10.1186/1742-2094-9-121

**Published:** 2012-06-07

**Authors:** Po-Kuan Chao, Kwok-Tung Lu, Ji-Yi Jhu, Yu-Yuan Peter Wo, Tai-Chun Huang, Long-Sun Ro, Yi-Ling Yang

**Affiliations:** 1 Department of Life Science, National Taiwan Normal University, 88, Section 4, Ting-Chou Road, Taipei, Taiwan; 2 Institute of Biochemical Science and Technology, National Chia-Yi University, 300, University Road, Chia-Yi, Taiwan; 3 Departments of Neurology, Chang Gung Memorial Hospital and Chang Gung University, 199 Tun Hwa North Road, Taipei, Taiwan

**Keywords:** Nogo-A, Traumatic brain injury, Inflammation, IL-1β

## Abstract

**Background:**

Nogo-A is a member of the reticulon family of membrane-associated proteins and plays an important role in axonal remodeling. The present study aimed to investigate alterations in Nogo-A expression following traumatic brain injury (TBI)-induced inflammation and neuronal damage.

**Methods:**

A weight-drop device was used to deliver a standard traumatic impact to rats. Western blot, RT-PCR and ELISA were used to analyze the expression of Nogo-A and IL-1β. Nogo-A antisense, and an irrelevant control oligonucleotide was intracerebroventricularly infused. We also performed H & E staining and luxol fast blue staining to evaluate the neuronal damage and demyelination resulting from TBI and various treatments.

**Results:**

Based on RT-PCR and western blot analyses, the expression of Nogo-A was found to be significantly upregulated in the hippocampus beginning eight hours after TBI. In addition, TBI caused an apparent elevation in IL-1β levels and severe neuronal damage and demyelination in the tested animals. All of the TBI-associated molecular and cellular consequences could be effectively reversed by treating the animals with the anti-inflammatory drug indomethacin. More importantly, the TBI-associated stimulation in the levels of both Nogo-A and IL-1β could be effectively inhibited by a specific Nogo-A antisense oligonucleotide.

**Conclusions:**

Our findings suggest that the suppression of Nogo-A expression appears to be an early response conferred by indomethacin, which then leads to decreases in the levels of IL-1β and TBI-induced neuron damage.

## Introduction

Traumatic brain injury (TBI) is one of the most prevalent causes of worldwide mortality and morbidity, and its treatment can result in enormous medical and social expenses [1,2]. Because of this, the World Health Organization (WHO) has ranked TBI as a 21st century epidemic with a severity equivalent to malaria and HIV/AIDS [3]. TBI causes neurological dysfunction and death through both primary and secondary cellular mechanisms. One of the primary effects is TBI-associated damage to axons, blood vessels, and glial cells in a focal or diffuse pattern. This damage might subsequently be amplified by certain secondary responses including hypoxia, hypotension, ischemia, edema, and intracranial pressure elevation [4]. Early studies have shown that the associated alteration in excitatory neurotransmitters, calcium overload, and reactive oxygen species (ROS) [5] also contribute to the development of TBI-induced primary and secondary damage.

The inflammatory response triggered by TBI was demonstrated to be closely related to TBI-induced neuronal cell death and functional deficits [6] and is characterized by glial activation, leukocyte recruitment, and upregulation of cytokine secretion [7,8]. In the list of the affected cytokines, IL-1 appears to be a key mediator of the TBI response. In fact, IL-1 has been reported to mediate many neurological effects in the brain [9]. A relatively high level of IL-1 has been found to be associated with TBI-induced neuron loss [7,10,11]. Thus, an efficient method that could ultimately confer a decline in IL-1 and the traumatic inflammatory response is likely to be an attractive strategy for TBI treatment [12,13].

Nogo-A, a myelin-rich membrane protein of the adult central nervous system (CNS), is known to act through specific binding to the Nogo receptor (NgR) [14]. Three isoforms of the Nogo protein (Nogo-A, Nogo-B, and Nogo-C) and of the corresponding NgRs have been identified [15]. The C-terminal sequences of all Nogo proteins bear a striking homology to several members of the reticulon or neuroendocrine-specific proteins, suggesting that Nogo-A is a member of the endoplasmic reticulum-anchored proteins. A growing body of studies has demonstrated that expression of Nogo-A is not restricted to neurons and oligodendrocytes in the CNS but occurs throughout the adult brain and spinal cord [16,17]. It is a potent inhibitor of neurite outgrowth, and it is known to negatively regulate regeneration in the adult CNS [18,19]. Treatment with anti-Nogo-A antibodies or an NgR receptor antagonist can significantly promote axonal regeneration, neuroanatomical plasticity, and functional recovery [20-22]. Furthermore, recent studies have also demonstrated that the expression of Nogo-A and NgRs is stimulated by the activated microglia/macrophages [23]. This converging evidence points to an important role for Nogo-A in mediating the inflammatory responses caused by various neurological conditions including TBI [24]. As the hippocampus was found to exhibit rather severe neuronal loss after TBI [7,25], in this study, we sought to investigate TBI-associated hippocampal Nogo-A expression, cytokine levels, and axonal and neuronal damage. In addition, we aimed to elucidate the correlation between Nogo-A production and post-TBI neuroinflammation using indomethacin.

## Methods

### Experimental animals

Adult male Wistar rats weighing 350 to 400 g were used in the current study. The rats were purchased from BioLASCO, Taiwan, Co., Ltd. and housed individually in hanging wire cages in a temperature-controlled animal colony at 24°C, with a normal 12-hour/12-hour light/dark cycle. The animals had free access to food and water, and they were allowed to acclimate to the light/dark cycle at room temperature for at least one week before undergoing the experiments. All animal experiment protocols were approved by the Animal Care and Use Committee of the National Chia-Yi University (Approval number: 100010). As a TBI model, a special weight-drop device which contained a foam bed on the bottom similar to that described by Marmarou *et al*. [26] was used to deliver a standard traumatic impact to the animals. Each rat was placed under pentobarbital anesthesia (40 mg/kg, i.p.), a midline incision was made on the head with a scalpel, and the skin flaps around the cutting site were peeled off laterally. After this, a metal helmet was sewn onto the top of the skull to prevent fracture from the trauma-inducing impact. Rats were then placed in a prone position on the bottom plate of the weight-drop device, and a 450-g weight was allowed to fall freely and vertically from a height of 1.8 m onto the metal helmet to induce TBI. In the experiments studying drug effects on the expression of Nogo-A and traumatic brain injury-associated inflammation and axonal damage, the rats were injected with Nogo-A  antisense   oligonucleotide  (5′-TGCTTTCGGTTGCTG AGGTA-3′) (i.c.v., 5 μl) [27] or indomethacin (i.p., 2.5 mg/kg, dissolved in 75% alcohol, Sigma, St. Louis, Missouri, USA) at the time of surgery while anesthetized.

### Nogo-A mRNA assay

The relative level of hippocampal Nogo-A transcription was determined by RT-PCR (n = 4, each group). After dissection of the brain, total hippocampal RNA was extracted with Trizol reagent (Gibco BRL, Grand Island, NY, USA), and 1 μg of each isolated RNA was subjected to cDNA synthesis. RT for cDNA synthesis was conducted in a 14 μL reaction buffer, containing 1 μL reverse transcriptase (50 U) and 1 μL oligo(dT)_15_ primer (50 pmol), according to the manufacturer’s instructions (Perkin Elmer, Foster City, CA, USA). The reaction was performed at 42°C for 30 minutes and subsequently terminated by boiling for 5 minutes. The obtained cDNA was then diluted to 100 μL with diethylpyrocarbonate (DEPC)-treated H_2_O, and the diluents were stored at −20°C prior to use. With the obtained cDNA (15 μL) as a template, the relative expression levels of Nogo-A from the animals receiving experimental treatment were determined by PCR. For PCR, a pair of specific primers, 5′-GCACAGCTTTGCCCATCA-3′ (forward) and 3′-GGCTTGTGCGACTCGA TCAC-5′ (reverse) (30 cycles), was used to amplify the Nogo-A gene, and another pair of primers, 5′-TGACGTTGACATCCGTAAAG-3′ (forward) and 3′-GAGATAGGACCGGAGTGACA-5′ (reverse) (28 cycles), was designed for the amplification of actin as an internal control [28]. The final PCR products were analyzed on an agarose gel, and the relative intensity was determined using semiquantitative densitometry in conjunction with AlphaEase software (Alpha Innotech Corp., Miami, FL, USA).

### Western blot analysis

The protein samples from various treatments were resolved by SDS-PAGE. The post-TBI rats (n = 6 in each group) were decapitated and the brains were removed at different time points after TBI. Following the dissection, the hippocampus was weighed and promptly homogenized in six volumes (v/w) of ice-cold homogenizing buffer, which contained 9.91 mM tris-base, 0.32 M sucrose, 1 mM ethylenediaminetetraacetic acid (EDTA), and proteases (PMSF: 100 μL/10 ml; leupeptin: 100 μL/10 ml; aprotinin: 100 μL/10 ml). Total proteins were fractionated on an 8% sodium dodecylsulfate polyacrylamide gel and the resolved proteins were electrophoretically transferred to a polyvinylidene difluoride (PVDF) membrane. The blotted membrane was then subjected to antibody detection. Polyclonal anti-Nogo-A (1:1,000 dilution; R&D System, Minneapolis, MN, USA) antibodies were used as primary antibodies, which were then detected by the secondary rabbit anti-goat antibody (1:5,000 dilution; Invitrogen, Grand Island, NY, USA) and visualized by an enhanced chemiluminescence assay (RPN 2108; Amersham International, Amersham, UK). We used actin as the internal control (1:5,000 dilution; Santa Cruz Biotechnology, Santa Cruz, CA, USA). Finally, the relative protein level of Nogo-A was quantified using semiquantitative densitometry equipped with AlphaEase software (Alpha Innotech Corp.).

### IL-1 detection

In our previous studies [7], we demonstrated that TBI could induce significant IL-1β overproduction and neuronal damage in the hippocampus and that the administration of an IL-1β antagonist could effectively protect animals from the trauma-associated damage. To elucidate the correlation between TBI-associated alterations in Nogo-A expression and the effects of indomethacin on IL-1β production, IL-1β expression was re-examined in this study by RT-PCR and ELISA. As in the prior study, the total RNA from the hippocampus of each rat was isolated for cDNA synthesis, and the obtained cDNA was used for PCR analysis (n = 6 in each group). The specific primers for IL-1β PCR amplification were 5′-ATGGCAACTGTTCCTGAACTCAAC-3′ (forward)    and    5′-AGGACAGGTATAGATTCTTTCCTTT-3′ (reverse)(30 cycles), and the primers for the actin internal control amplification were 5′-TGACGTTGACATCCGTAAAG-3′ (forward) and 5′-ACAGTGAGGCCAGG ATAGAG-3′ (reverse) (28 cycles) [27]. The subsequent analysis procedure assessing the PCR product for IL-1β expression was similar to that for the Nogo-A determination. The concentration of IL-1 was also measured using a commercial ELISA kit according to the manufacturer’s instructions (Bender Medsystems, San Diego, CA, USA).

### Water content measurement

Rats (n = 4 in each group) were decapitated under deep anesthesia with 100 mg/kg pentobarbital. The brains were quickly removed and their wet weights were measured. The tissue was dried at 120°C for 24 hours. In a double-blinded manner, the water content was calculated as the difference between wet and dry weight and expressed as the percentage of wet weight [25].

### Evaluation of neuron damage: H & E staining

Three days post-TBI, each group of rats was sacrificed using an overdose of pentobarbital (100 mg/kg, i.p.) and then perfused transcardially with 0.9% NaCl and 10% formalin. After perfusion, the rats were decapitated and their brains were removed and embedded in paraffin blocks (n = 5 in each group). Coronal sections (10 μm thickness) were stained with H & E and subjected to microscopic examination [7,25].

### Evaluation of axonal demyelination: Luxol fast blue staining

Using a similar procedure to the one described above, the axonal damage was also analyzed. The embedded coronal sections (10 μm thickness) were stained with luxol fast blue (LFB, 0.1%, Sigma) and cresyl echt violet (0.1%, Sigma) for myelin detection and axonal loss assessment (n = 5 in each group).

### Statistical analysis

The obtained data are presented as the means ± standard error of the mean (SEM). Kruskal-Wallis analyses of variance (ANOVA) were conducted, and if significant, were followed by the Mann–Whitney *U* test. *P* <0.05 was considered statistically significant.

## Results

### Upregulation of Nogo-A after TBI

**Figure 1:**
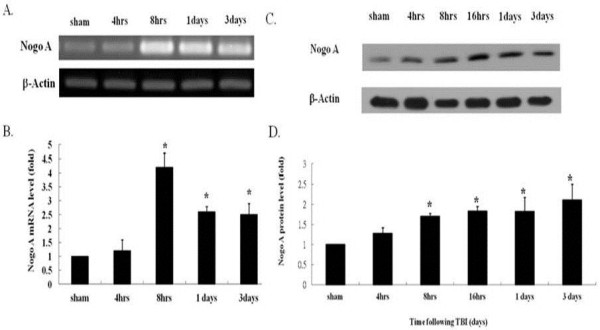
**RT-PCR analysis and western blot analysis of Nogo-A mRNA and protein production in the hippocampus after TBI. (A)** The PCR products from Nogo-A transcription after TBI with the yield of actin as an internal control. The sampling times after TBI are shown on the top (n = 4 in each group). **(B)** Quantification of Nogo-A mRNA expression by semiquantitative densitometry in conjunction with AlphaEase software (Alpha Innotech Corp.). **(C)** Time course of Nogo-A protein production after TBI, internal control: β-actin. Times shown on top represent hours after injury. **(D)** Quantification of Nogo-A protein by semiquantitative densitometry in conjunction with AlphaEase software (Alpha Innotech Corp.). The data are presented as ratios related to the sham group. Bars represent the means ± SEM values. *p < 0.05 is considered significantly different from sham values using the Mann–Whitney *U* test. SEM, standard error of the mean; TBI, traumatic brain injury.

**Figure 2:**
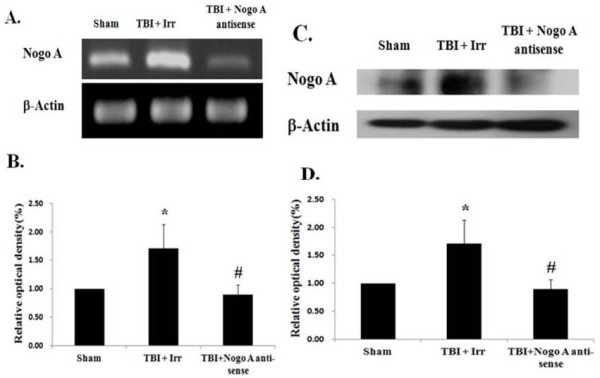
**Effects of Nogo-A irrelevant control and antisense oligodeoxynucleotides on hippocampal Nogo-A expression after TBI. (A)** RT-PCR analysis of Nogo-A mRNA transcription level. Actin transcription was used as an internal control. **(B)** The expression of Nogo-A was quantified by densitometry and compared with the data from rats injected with saline (sham), which was normalized to 100%. **(C)** Western blot analysis of Nogo-A protein level; β-actin was used as an internal control. **(D)** Quantification of Nogo-A protein by semiquantitative densitometry in conjunction with AlphaEase software (Alpha Innotech Corp.). The data are presented compared with the sham group. The data are represented as the means ± SEM values (n = 6). *p < 0.05 was considered significantly different from the sham value using the Mann-Whiney *U* test, and ^#^*p* < 0.05 was considered significantly different from the TBI with sense values using the Mann-Whiney *U* test. SEM, standard error of the mean; TBI, traumatic brain injury.

The first experiment conducted in the current study sought to examine alterations in the expression of Nogo-A in the hippocampus after TBI. Compared with the sham group, the Nogo-A mRNA expression level was found to rise slightly at four hours after TBI induction, but this difference was not significant (Figure 1A, B). The upregulation of Nogo-A expression reached a maximum at eight hours after trauma (approximately four times the maximum level in the sham group, *P* <0.05) and lasted for three days. This stimulatory effect on Nogo-A production was further confirmed by protein analysis. Western blot analysis revealed an increase in Nogo-A protein in the hippocampus four hours post-TBI. However, a statistically significant elevation n the protein level began at eight hours after TBI and lasted for three days (Figure 1C, D). Moreover, this TBI-induced stimulation of Nogo-A expression could be reversed by the administration of Nogo-A antisense oligonucleotide (5′-TGCTTTCGGTTGCTGAGGTA-3′) immediately after TBI. As shown in the RT-PCR analysis (Figure 2A and B) and western blot analysis (Figure 2C and D), microinjection of Nogo-A antisense oligonucleotide into the lateral ventricle drastically decreased the TBI-induced Nogo-A production by approximately 70%. However, the Nogo-A irrelevant control oligonucleotide (5′-GCAGACCAGCGCGGA GCT-3′) appeared to be ineffective in decreasing the TBI-associated Nogo-A production.

### Indomethacin attenuated expression of Nogo-A

**Figure 3:**
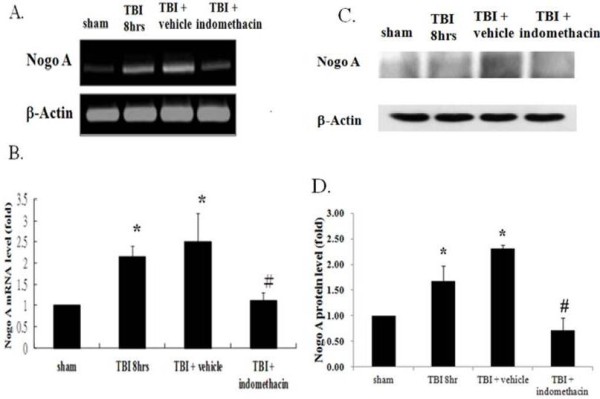
**Effects of indomethacin administration on Nogo-A expression.** Animals were in one of four groups: sham (no TBI), TBI treatment (TBI eight hours), TBI combined with vehicle administration (TBI + vehicle), and TBI combined with indomethacin administration (TBI + indomethacin). **(A)** RT-PCR analysis of the expression of Nogo-A among different groups along with the analysis of β-actin transcription as an internal control. **(B)** Quantification of Nogo-A expression. **(C)** Western blot analysis of the expression of Nogo-A among different groups along with the analysis of β-actin as an internal control. **(D)** Quantification of Nogo-A expression. Bars represent means ± SEM values (n = 5). *P <0.05 is considered significantly different from the sham value using the Mann–Whitney *U* test and #*P* <0.05 is considered significantly different from the TBI value using the Mann–Whitney *U* test. SEM, standard error of the mean; TBI, traumatic brain injury.

**Figure 4:**
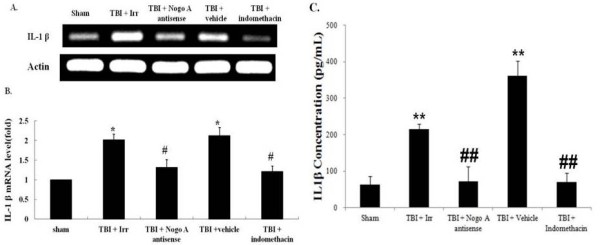
**Effects of TBI, Nogo-A irrelevant control oligonucleotide, Nogo-A antisense oligonucleotide, and indomethacin administration on the production of IL-1β.** All samples were obtained eight hours after TBI. The relative level of the released IL-1β is compared among different treatment groups: neither TBI nor drug (sham); TBI and Nogo-A irrelevant control oligonucleotide (TBI + Irr), TBI and Nogo-A antisense oligonucleotide (TBI + Nogo-A antisense); TBI and solvent vehicle (75% alcohol) (TBI + vehicle); and TBI combined with indomethacin injection (TBI + indomethacin). **(A)** RT-PCR analysis of the expression of IL-1β among different groups along with the analysis of actin transcription as an internal control. **(B)** Quantification of IL-1β expression. **(C)** ELISA analysis on the expression of IL-1β protein levels among different groups. Bars represent the means ± SEM values (n = 5). *P <0.05 is considered significantly different from the sham value using the Mann–Whitney *U* test, and #*P* <0.05 is considered significantly different from the TBI-eight hour value using the Mann–Whitney *U* test. SEM, standard error of the mean; TBI, traumatic brain injury.

Indomethacin, a potent non-steroidal anti-inflammatory drug, was used in this experiment to determine the relationship between TBI-associated inflammatory effects and Nogo-A expression. The level of Nogo-A was again significantly increased as a consequence of TBI, whereas in the TBI rats that were given indomethacin, Nogo-A expression at both the mRNA (Figure 3A and B) and protein (Figure 3C and D) levels returned to those observed in sham animals. Unlike the direct effect conferred by Nogo-A antisense oligonucleotide, indomethacin may conceivably have triggered a novel pathway that resulted in the suppression of Nogo-A expression. However, an interesting finding was that the administration of indomethacin or Nogo-A antisense, but not Nogo-A irrelevant control oligonucleotide, not only suppressed the Nogo-A overproduction but also downregulated the expression of IL-1β mRNA (Figure 4A and B) and protein (Figure 4C) after TBI. This strongly suggests that TBI-induced IL-1β production is modulated by the level of Nogo-A.

### Administration of Nogo-A antisense and indomethacin protects animals from TBI-induced brain edema, neuronal damage, and demyelination

**Figure 5:**
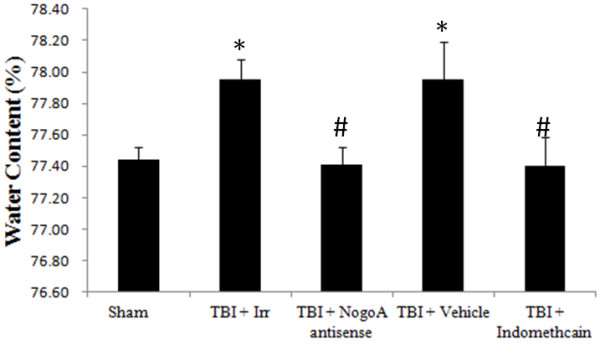
**Effects of indomethacin and Nogo-A antisense administration on brain edema following traumatic brain injury (TBI).** Animals were divided into five groups: neither TBI nor drug (sham); TBI and Nogo-A irrelevant control oligonucleotide (TBI + Irr); TBI and Nogo-A antisense oligonucleotide (TBI + Nogo-A antisense); TBI and solvent vehicle (75% alcohol) (TBI + vehicle); and TBI combined with indomethacin injection (TBI + indomethacin). Bars represent the means ± SEM values (n = 4). *P <0.05 was considered significantly different from the sham values using the Mann–Whitney *U* test; #*P <*0.05 was considered significantly different from the TBI values using the Mann–Whitney *U* test. SEM, standard error of the mean; TBI, traumatic brain injury.

**Figure 6:**
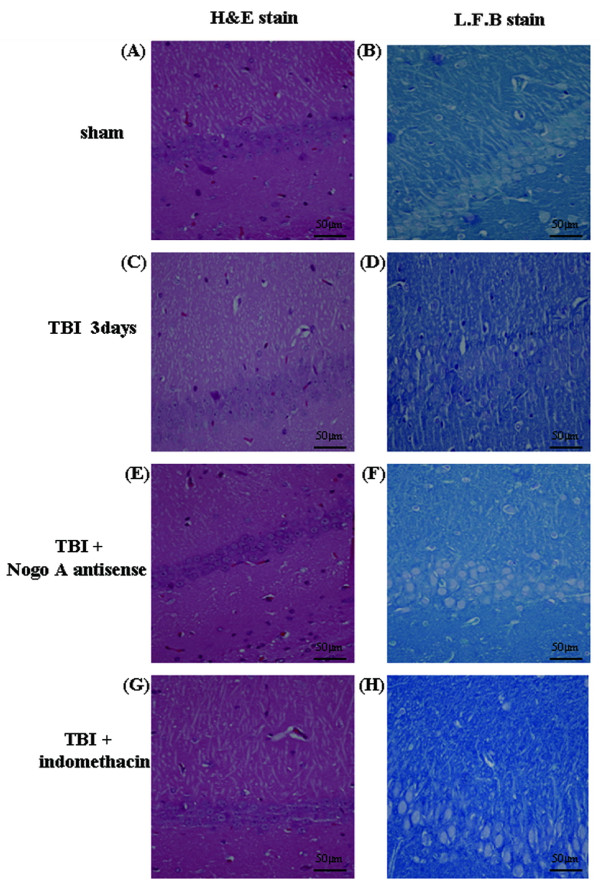
**Indomethacin and Nogo-A antisense treatment protects animals from TBI-induced neuronal death and axonal demyelination.** The neurons from animals in different groups: (**A, B**) rats without TBI (sham); (**C, D**) TBI only; (**E, F**) TBI combined with the administration of Nogo-A antisense (TBI + Nogo-A antisense); (**G, H**) TBI combined with indomethacin administration (TBI + Indomethacin). The coronal sections (10 μm thickness) through the hippocampus were stained with either H & E or luxol fast blue (LFB) for microscopic analysis to determine the neuronal damage or axonal demyelination, respectively (n = 5 in each group). TBI, traumatic brain injury.

In our previous study, we found that TBI induced severe brain edema. In this study, we attempted to evaluate the effect of Nogo-A antisense and indomethacin on TBI-induced brain edema formation. TBI-induced neuronal damage and demyelination were analyzed by H&E and luxol fast blue staining, respectively. Compared with the sham group, TBI indeed led to severe brain edema, neuronal damage, and demyelination, as indicated by neuronal swelling, shrinkage, and subsequent neuronal loss (Figures 5, 6A-D). This TBI-associated brain edema and damage could be effectively diminished by the administration of Nogo-A antisense oligonucleotide (Figures 5, 6E and 6F) or indomethacin (Figures 5, 6G and 6H). The results again suggest that the complicated neuroprotective mechanism against TBI-induced damage elicited by indomethacin should be at least in part mediated by Nogo-A. Additionally, as described above, the change in the level of IL-1β is modulated by that of Nogo-A, suggesting that the alteration of Nogo-A expression might be an early stage event in the protection process conferred by indomethacin.

## Discussion

Our results demonstrate that the production of Nogo-A mRNA and protein is stimulated several hours after TBI in the hippocampus, and such TBI-induced upregulation of Nogo-A can be suppressed by treatment with indomethacin. The increase in the levels of IL-1β and the TBI-associated demyelination and neuronal damage could also be effectively reversed by this non-steroidal anti-inflammatory drug. More interestingly, the expression of Nogo-A was found to be well-correlated with hippocampal IL-1β release, as blockage of Nogo-A by an antisense oligonucleotide could prevent IL-1β from overloading. These results suggest that the neuroprotective activity of indomethacin is mediated by the repression of Nogo-A expression in the early stages of the process. Subsequently, the downregulated Nogo-A then promotes a decline in the release of IL-1β via a pathway that is yet to be characterized.

Our results on the profile of Nogo-A expression in an adult rat TBI model are consistent with those observed in neonatal rat middle cerebral artery occlusion (MCAO) or pyramidal tract lesion models [29,30], but differ from previous observations in adult rat MCAO models [31]. In the neonatal ischemic rats, Nogo-A expression peaked within 24 hours and returned to near baseline level by 72 hours [29] whereas in the adult rats, MCAO caused an alteration in neuronal Nogo-A expression continuously in the ipsilateral and contralateral cortex and conferred a global elevation at 28 days after stroke [31]. As we observed, the expression of Nogo-A reached a plateau at 8 hours post-TBI and lasted for 72 hours (three days), which is not entirely consistent with either of the above models. We suspect this may be due to the differing procedures adopted in brain injury induction. In our model, the TBI-induced diffused damage may not be restricted to neuronal injury. It might also cause vessel ruptures and severe damage to the blood brain barrier (BBB). Consequently, TBI could have accelerated the recruitment of microglia and macrophages, and in turn promptly stimulated earlier Nogo-A expression. The other possible explanation for the variations in Nogo-A expression profiles may be the specific brain area that was subject to investigation in this study. Based on the results from our previous studies showing that the hippocampus exhibits rather severe neuronal loss due to TBI [7,25], it follows that a more rapid TBI response, including Nogo-A upregulation, should be more clearly observed in the hippocampus.

The role of Nogo-A is controversial. Using monoclonal antibodies (mAbs) to neutralize Nogo-A or using soluble fragments of NgR to block the Nogo-A-NgR interaction has been found to increase axonal outgrowth and sprouting significantly, which correlates with an improvement of functional outcome after cerebral ischemia or stroke [22,32,33]. Neutralization of Nogo-A by mAbs has also been shown to improve cognitive recovery after TBI [34]. These studies suggest that Nogo-A elicits the axonal inhibitory effects after injury and provide a potential treatment strategy for TBI. However, contrary to the pharmacological results, the genetic deletion of Nogo-A did not improve functional and histological outcomes after TBI in aging animals [35]. Compared with wild littermates, the Nogo-A/B deficient mice showed diminished recovery from neurological motor deficits and reduced area and density in the corpus callosum after TBI [35]. In addition to these interesting results observed in the Nogo-A/B deficient mice, the NgR deficient mice also display impaired cognitive outcomes in the Morris water maze task after TBI [36]. It has also been reported that Nogo-A plays a critical role in stabilizing and maintaining the architecture of hippocampal pyramidal neurons [37]. These results suggest that the role of Nogo-A in TBI-induced neuronal damage is very complex and may also be age-dependent.

Brain edema is one of the characteristic features observed in patients suffering from severe traumatic brain injury, and it can be classified into vasogenic edema and cytotoxic edema. Vasogenic edema, a secondary response to BBB compromise following TBI, can lead to a swelling process and a subsequent rise in intracranial pressure. Cytotoxic edema causes intracellular fluid accumulation and occurs during water intoxication and under anoxia-generating conditions, such as trauma and stroke [25]. Earlier studies have demonstrated that some neuroprotective agents, such as tamoxifen, could protect animals from spinal cord injury-induced edema and neuronal damage via the attenuation of Nogo-A [38]. Some researchers also found that Nogo-A could also trigger a rapid phosphorylation of the epidermal growth factor receptor and subsequently activate a MAPK signaling pathway via the phosphorylation of MEK and ERK [39]. A similar ERK/MEK/Raf cascade activation was also observed in our previous studies on TBI-induced brain edema [25]. The TBI-associated stimulation in Nogo-A might have provided a connection that correlates the MAPK pathway to the TBI-induced cytotoxic brain edema. Indomethacin administration significantly reduced the intracranial pressure [40,41] and BBB disruption [42], which may attenuate vasogenic brain edema [43]. The protective effect of indomethacin is speculated not only to reduce the vasogenic edema that results from TBI-induced intracranial pressure elevation but also to attenuate cytotoxic edema through the inhibition of the Nogo-A/MAPK pathway.

A large number of studies have indicated that inflammation is important to TBI-induced secondary damage to neurons, glia and myelin [44,45]. TBI induces the rupture of the BBB and various inflammatory responses, including cytokine release, the accumulation of leukocytes, and activation of macrophages and microglia [7,8,46]. Prostanglandin E2 (PGE2) is one of the early inflammatory mediators released by macrophages. Several studies have demonstrated that PGE2 is significantly elevated in the plasma of traumatized patients and animals [45,47,48] and is important for macrophage activation; macrophages may migrate toward the site of injury, secrete toxic cytokines, and thereby cause further neuronal damage [44,49]. Indomethacin, a non-specific cyclooxygenase inhibitor, reduces PGE_2_ production and elicits a potent anti-inflammatory effect. In this study, we found that indomethacin treatment significantly attenuated the TBI-induced elevation of hippocampal Nogo-A and IL-1β. Recent studies have indicated that Nogo-A receptors are expressed in macrophages in injured peripheral nerves [23] and in oligodendrocytes of the central nervous system [19]. It is highly possible that indomethacin blocks PGE_2_ production, which then attenuates the activation of macrophage/microglia and further reduces the expression of Nogo-A and IL-1β release. However, further study is needed to verify and delineate this complex mechanism.

## Conclusions

The results presented here indicate that Nogo-A plays an important role in TBI-induced IL-1β release and neuronal and axonal damage. By inhibiting Nogo-A expression, the systemic delivery of indomethacin can greatly ameliorate the TBI-induced IL-1β overload and neuronal damage.

## Abbreviations

BBB, Blood brain barrier; ERK, Extracellular signal-regulated kinase; IL-1, Interleukin-1; MAPK, Mitogen-activated protein kinase; PCR, Polymerase chain reaction.

## Competing interests

The authors declare that they have no competing interests.

## Authors’ contributions

P-KC and K-TL contributed equally to this work. P-KC established the TBI model and helped J-YJ to analyze the expression of Nogo A and cytokine. J-YJ performed the Western blot analysis, PCR, and histological evaluation. T-CH performed the brain edema experiments. Y-Y-PW helped to analyze and interpret the PCR results and in manuscript editing. L-SR provided the statistic analysis consulting. K-TL and Y-LY secured the funding for the project and helped with the final version of the manuscripts. All authors have read and approved the final manuscript.
